# Double layer steganography technique using DNA sequences and images

**DOI:** 10.7717/peerj-cs.1379

**Published:** 2023-05-16

**Authors:** Asia Othman Aljahdali, Omnia Abdullah Al-Harbi

**Affiliations:** 1 Cybersecurity Department, College of Computer Science and Engineering, University of Jeddah, Jeddah, Saudi Arabia; 2 Department of Computer Science and Artificial Intelligent, College of Computer Science and Engineering, University of Jeddah, Jeddah, Saudi Arabia

**Keywords:** Steganography, DNA sequences, Stego image, PSNR

## Abstract

Information security has become increasingly challenging as a result of massive advancements in information and communication technologies. Due to the necessity of exchanging private information and the open nature of the network, there is an increased risk of various types of attacks. Consequently, data security is an essential component of data communication. One of the most effective methods used to achieve secrecy is steganography. This method hides data within a cover object without raising suspicion. The level of security is improved when two steganography methods are combined. This approach is known as multilevel steganography, which hides sensitive data in two cover objects in order to provide a two-level security system. Accordingly, we developed a technique that focuses on protecting secrecy while also being robust to attacks. The new technique uses a multi-layer steganography mechanism by using DNA sequences and images as carriers for sensitive data. The technique intends to hide secret messages in the DNA using the substation algorithm, and then the fake DNA is embedded in an image utilizing the discrete cosine transform (DCT) method. Eventually, the stego image is sent to the intended recipient. Different types of images with different sizes and lengths of messages and DNA sequences were used during the experiments. The results show that the proposed mechanism is resistant to histogram and chi-square attacks. The maximum mean value observed was 0.05, which means the histograms of the original and stego images are nearly identical, and the stego image does not raise any suspicion regarding the existence of secret information. In addition, the imperceptibility ratios were good, as the highest PSNR and MSE values were 0.078 and 72.2, respectively. Finally, the PNG and BMP images show excellent results. On the other hand, the JPG images failed to meet the expected ratio of imperceptibility and security.

## Introduction

The significant improvement in information and communication technologies over the last few decades has increased information security challenges. The security of the data during transmission is essential. The need to exchange sensitive information, as well as the network’s openness, increases the probability of numerous attacks. Therefore, data security is important in areas of data communications (Vinodhini & Malathi, 2018).

The two most popular and significant mechanisms for achieving secrecy are cryptography and steganography. Although these processes differ, they both ensure data security, confidentiality, and integrity (Taha et al., 2019). Cryptography is a collection of methods based on mathematical concepts. It alters intelligible information into a meaningless form that is unreadable to unauthorized individuals. On the other hand, steganography allows for undetectable communication by concealing data in a cover object (Taha et al., 2019). Cover objects such as text files, images, videos, and DNA sequences are used to hide the hidden data. Steganography’s main intention is to conceal sensitive information to avoid suspicion (Tayana, 2013). It relies on hiding the existence of secret data from individuals. In comparison, cryptography offers the ability to transmit the data in a way that prevents unauthorized individuals from reading it.

A two-layer of security is provided by combining cryptography and steganography. The cryptography scrambles the secret message, and then steganography hides the encrypted data in a cover object (Aung & Naing, 2014). In the same way, a combination of two steganography methods improves the level of security. This approach is called multilevel steganography, which relies on hiding the sensitive data in two cover objects in order to provide a two-level of security.

As mentioned, the security of data is a real problem when transmitting secret data to remote users over an insecure channel. Accordingly, this research aims to develop a technique that concentrates on protecting secrecy, while paralleling being robust to attacks. The new technique uses a multi-layer steganography mechanism by using DNA sequences and images as carriers for sensitive data. The algorithm of the technique intends to hide secret messages in the DNA, and then the fake DNA is embedded in an image by using an embedding method. Eventually, the result of this algorithm is a stego-image that is sent to the intended recipient.

The proposed technique can be used in various domains, including securing the data from enemies, communicating sensitive data such as criminal records, or transferring patients’ data. The main contributions of this study are

1. Develop a technique for data hiding that concentrates on protecting secrecy while being robust to attacks using DNA sequences.
2. Analyze the effectiveness of the proposed system using different types of images with different sizes and lengths of messages and DNA sequences using several metrics.
3. Analyze the security and robustness of the proposed system.

The following is a breakdown of the article’s structure. Section 2 provides an overview of DNA-based and image-based steganography. Section 3 discusses the related works that have been reviewed. Section 4 illustrates the proposed technique and the methods used to develop it. Section 5 is about the experiments and the evaluation metrics that were used, and the results of these experiments, besides comparisons with existing techniques. Section 6 concludes with a review of the anticipated work.

## Background

This section begins with a review of the steganography concept, followed by a general biological overview of DNA, and concludes with the use of DNA as a cover object in steganography. It also discusses image-based steganography and its various types.

### Steganography

Steganography is defined as a set of mechanisms that are concerned with concealing secret data in a cover object in such a way that the hidden data is invisible to individuals (Cox et al., 2007). The term steganography comes from the Greek word “covered writing”. Steganography cFan hide various types of data within a cover file, like text, images, videos, or anything that can be represented as a bit-stream. Moreover, there are different types of cover objects, such as text files, images, videos, and DNA sequences.

Mathematically, the below equations represent the embedding (Emb) and extraction(Ext) processes. Let c represents a cover object from a set C of all cover objects, k denotes an optional key from a set K of all keys, *C*′ is stego image, and m indicates a secret message from a set M of all secret messages (Cox et al., 2007).

(1) \begin{eqnarray*}Emb:C{}K{}M\rightarrow {C}^{{}^{{^{\prime}}}}\end{eqnarray*}

(2) \begin{eqnarray*}Ext:(EM(c,k,m))=m,for\,all~c\in C,k\in K,m\in M.\end{eqnarray*}

**Figure 1:**
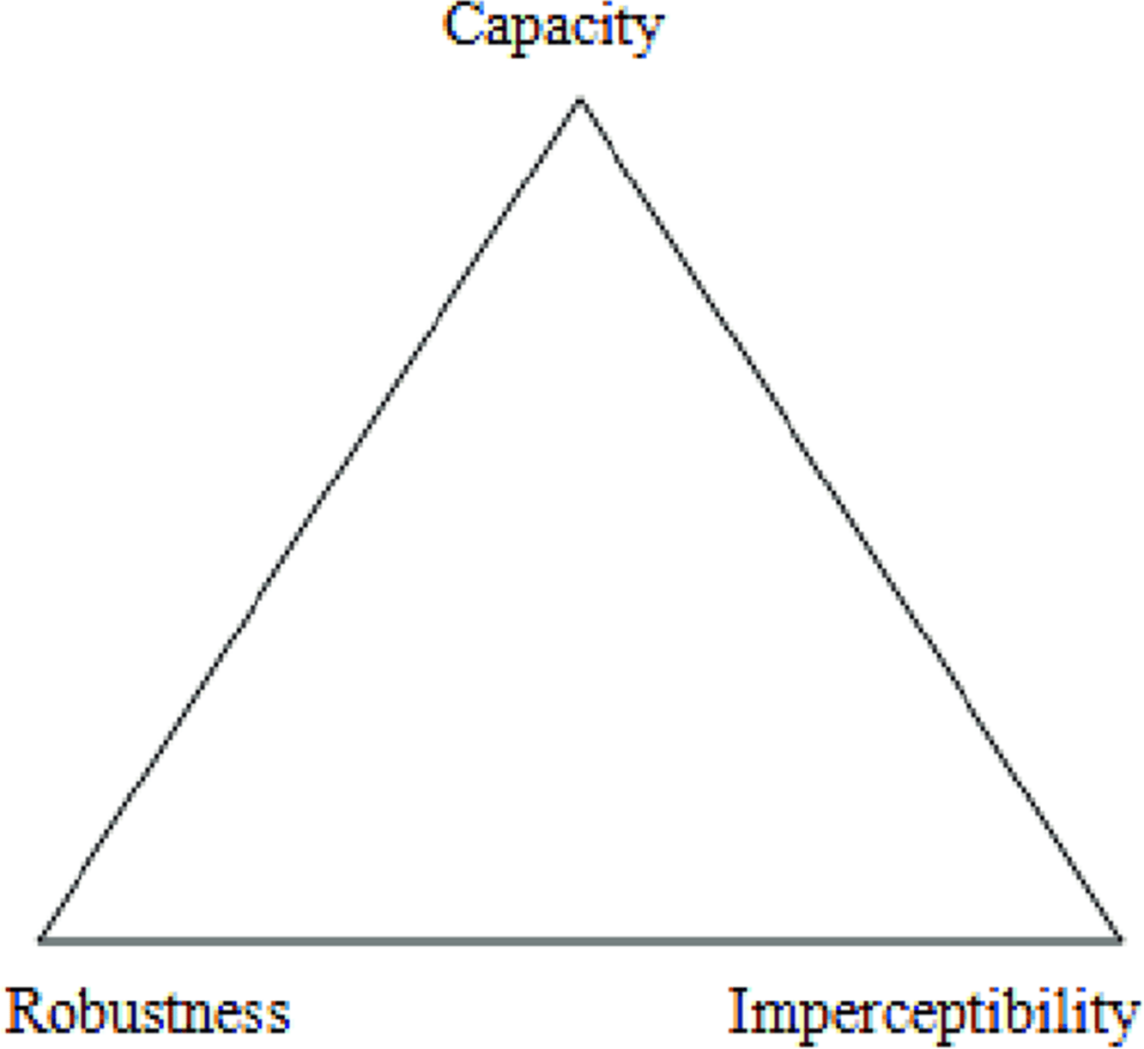
Steganography triangle (Majeed et al., 2021).

Any steganography technique’s main objective is to provide safe, useful, and efficient communication that is also completely undetectable. Any object with a lot of redundant bits is therefore considered perfect since it is tough to determine if a redundant bit has changed (Hmood et al., 2010). The redundant bits of a cover object that give precision considerably greater than needed for the object’s use and presentation are referred to as redundancy in this context. As a result, a safe steganography technique is one in which the original cover item cannot be distinguished from the stego object by a person or computer. The effectiveness of any steganography algorithm is measured using three criteria. The first is capacity, which refers to the maximum quantity of hidden information that an object can contain (Altaay, Sahib & Zamani, 2012). The second criteria is imperceptibility, which states that the stego-cover and original cover are identical and cannot be distinguished from one another (Altaay, Sahib & Zamani, 2012). Third, robustness, which is the resistance to attacks and unexpected modifications (Kaur, Bansal & Bansal, 2014). The association between these three criteria is represented by the steganography triangle shown in Fig. 1 (Majeed et al., 2021). In the end, it is vital to maintain the robustness while expanding the capacity and imperceptibility.

### DNA

#### Biological overview

Deoxyribonucleic acid, or DNA, is a molecule that houses the genetic information required for the growth and operation of all recognized living things, including viruses (Hamed et al., 2016). Four chemical bases—adenine (A), guanine (G), cytosine (C), and thymine (T)—collectively known as nucleotides are used to encode the information found in DNA (Hamed et al., 2016).

#### DNA based steganography

Deoxyribose nucleic acid (DNA) has been used to perform computation operations and is considered a branch of computing called DNA computing. DNA sequences have various biological properties that can be employed for solving intractable problems (Ibrahim, Rashid & Sadiq, 2016). Therefore, the biological properties of DNA sequences are exploited to obtain secure and robust cryptography and steganography techniques.

The scientific discipline of DNA steganography is a recent and cutting-edge one that began in 1999. For a number of reasons, the DNA sequences served as an effective transporter for the secret information. A gram of DNA can hold approximately 108 terabytes, therefore, it has a large storage capacity (Hamed et al., 2016). Additionally, the complexity and randomness of DNA sequences provide dependable security, robustness, and protection (Guo, Chang & Wang, 2012). Furthermore, the easiness with which data may be changed into a DNA sequence and vice versa. Eventually, original DNA and altered DNA cannot be distinguished from one another (Marwan, Shawish & Nagaty, 2017).

A common and easy way of translating data into a DNA format and vice versa is to translate each DNA base into two binary bits. Table 1 displays how DNA is represented in binary. Some of the methods nevertheless developed their own conversion rules.

**Table 1:** DNA-binary representation.

DNA base	Binary representation
A	00
C	01
G	10
T	11

### Image steganography

Due to their widespread use online, images are the type of cover object utilized most frequently in steganography. It is regarded as the ideal cover object because of the high quantity of superfluous bits. Image steganography techniques can be divided into two categories: the transform domain and the spatial domain.

The spatial domain methods directly alter the image by changing some of the image’s pixel values with the secret message bits (Hussain & Hussain, 2013). This approach makes use of the huge amount of redundant data. Here are some examples of spatial domain methods: spatial domain methods: least significant bit (LSB), pixel value differencing (PVD), edges based data embedding method (EBE), and random pixel embedding method (RPE).

The transform domain method is another name for the frequency domain method. Before embedding the secret message, these approaches require transforming the cover-image from the spatial domain to the frequency domain (Hussain & Hussain, 2013). This approach hides messages in significant areas of the cover image, which makes it more resistant to image manipulation attempts. Frequency domain methods include the discrete fourier transformation technique (DFT), the discrete cosine transformation technique (DCT), and the discrete wavelet transformation technique (DWT).

## Literature Review

Because the combination of image-based and DNA-based steganography is so new, there are not many works that deal with concealing data using this approach. As per literature review done by Alharbi, Aljhadli & Manaf (2020), Table 2 shows the contribution of researchers in this field.

Vinodhini & Malathi (2018) The technique depends on splitting the DNA sequence into equal segments, and after that, the secret data is inserted using the insertion method at the end of each segment. The developers tried two distinct approaches for hiding the fake DNA sequence inside the cover image, LSB and F5. The results show that the LSB approach is easily exploitable, and that the fake DNA sequence is easily recoverable. F5 methods, on the other hand, provide superior security.

Roy et al. (2015) employs the insertion technique to conceal the secret info within the DNA sequence Following that, the DNA sequence is encrypted. The LSB technique is then used to conceal the encrypted DNA sequence within an image.

Das et al. (2015) uses a fake DNA sequence rather than a real one, by creating it from the cover image. Using the replacement method, the secret data is entered into the DNA. The two-bit least significant bit (LSB), used to conceal two bits in a single pixel, is then used to insert the mutated DNA into the cover image.

**Table 2:** Comparison between the related works.

Criteria	DNA hiding technique	Image hiding technique	Strength	Limitation
Malathi et al. (2017)	Insertion Method	LSB F5	Uses the F5 algorithms for hiding the DNA sequence into image	Using the insertion method.
Roy et al. (2015)	Insertion Method	LSB	Authentication mechanism and Encrypting the DNA sequence	Using the insertion and LSB methods
Das et al. (2015)	Replacement Rules	LSB	Encrypting the secret data using RC4 before the hiding process	Two-bit least significant substitution (LSB)
Tuncer & Avci (2016)	XOR	XOR	The best PSNR values among the others and it uses the DNA-XOR and LSB together for hiding the data	Complexity of the algorithm
Durafe & Patidar (2022)	Substitution and XOR	DWT-SVD embedding techniques	The secret image is secured with dual layers of encryption using DNA encoding and a standard chaotic map.	The cover image is only in PNG format, with dimensions of 512 ×512

For Tuncer & Avci (2016), the data is hidden in the DNA sequence using the DNA-XOR approach. And, using LSB, each bit of the fake DNA is placed in the cover image’s red, green, and blue channels, respectively.

Durafe & Patidar (2020) proposes a method based on fractal cover images, singular value decomposition (SVD), integer wavelet transform (IWT), and discrete wavelet transform (DWT). The main concept is to hide the secret color image behind a fractal cover image. These fractal images are generated using unique mathematical equations identified by a set of fractal parameters. To increase security and robustness, authors make use of SVD and DWT or SVD and IWT. The algorithm has greater hiding capacity due to fractal compression, DWT-SVD, and IWT-SVD hybrid transform schemes. The experimental results show that image degradation is less than 0.6%.

A study in Durafe & Patidar (2022) develops an efficient and robust blind steganography model by integrating fractal cover images, a standard chaotic map, DNA-chaotic encryption, and the DWT-SVD embedding method. The secret image is encrypted using DNA encoding and a standard chaotic map, which is used to generate the pseudo-random number sequences of cryptographic qualities. Global bit scrambling (GBS) is utilized to improve image encryption performance. In order to reconstruct the cover image for extraction, only a unique fractal key is shared with the receiver. The secret image is encrypted using the S-map then with the hyperchaotic sequence in conjunction with the DNA sequence. GBS is used for permutation and substitution of the pixel values along with DNA addition, complement, and bit XOR thus enhancing the sensitivity of the image. The DNA-chaotic combination offers dual encryption security to the secret information, and the chaotic behavior is validated using the NIST tests.

As a conclusion, the DNA sequence in Das et al. (2015) and Tuncer & Avci (2016) is made from either a cover image or secret data and is not real. The secret message bits are inserted into the DNA sequence at different points in Malathi et al. (2017) and Roy et al. (2015), which lengthens the DNA sequence. This technique, known as the insertion method, is classified as the most overt data hiding technique that attracts unauthorized users’ attention. In Roy et al. (2015) and Das et al. (2015), the Least Significant Bit (LSB) method had been employed, which was considered the simplest embedding method for concealing hidden information within an image.

## Methodology

After reviewing the work done on multilevel steganography using DNA sequences and images together as cover objects, the main contribution is introducing a new technique that focuses on avoiding the weaknesses of the reviewed work and achieving security. Moreover, to improve efficiency, other criteria were identified and employed during the development of the proposed work, which includes utilizing DCT for embedding the image and using different image types and sizes to evaluate the proposed method. For the steganography method, the substitution method is selected since it provides security, high capacity, and a payload equal to zero, as shown in Table 3.

To achieve our aims, we have to avoid the limitations of the reviewed work. The following are the most important aspects that must be complied with:

• Using a real DNA sequence to get the benefits of its complexity and randomness gives high protection, security, and robustness.
• Making use of an effective and secure DNA steganography method.
• Using an efficient image steganography method (DCT) that gives a high visual quality stego-image to avert attackers’ suspicion.

We can describe the proposed work as a layered architecture that consists of two layers or stages. Each layer performs a particular role, and it must go through the layers in sequential order. The architecture’s input is a secret message that must be hidden inside a cover object in order to be invisible to unauthorized individuals. The output is an image that contains the secret message. The next sections will describe these layers and the methods used in each one in detail.

The proposed technique is implemented using the Conda environment in a Jupyter notebook and using the Python language, the full code is in Alharbi (2022); it consists of two main functions as below:

1. The secret information is embedded into a real DNA sequence using the substitution algorithm with the use of a pseudo-random generator (PRG). Subsequently, the fake DNA that contains the secret message will be embedded into an image using the discrete cosine transform (DCT).
2. The extracting mechanism: is similar to the embedding mechanism, in which the fake DNA is retrieved from the steg-image and the secret message is retrieved from the fake DNA.

### DNA-based steganography Layer

The goal of DNA-based steganography is to strengthen security by using a real DNA sequence, because the complexity and randomness of real DNA offer excellent protection, security, and robustness. To embed the hidden message in DNA-based steganography, three primary methods are used: insertion, substitution, and complementary rule-based methods (Shiu et al., 2010).

Table 3 shows a comparison between them (Zebari, Haron & Zeebaree, 2017). For the insertion method, the DNA sequence length will increase as the secret message is added to it. Because of that, the payload does not equal zero. Additionally, both the sender and the receiver must agree on which part of the message will be inserted, so it is an unblinded algorithm. For the complementary pair rules, both the sender and the receiver must agree on the rules before starting the process, and it’s considered a secure method because the attacker has to know the rules to extract the data unless the attacker eavesdrops on the prior communication between the two sides.

**Table 3:** DNA-based steganography methods.

Method name	Description	Strength points	Weakness
Insertion	DNA sequence is segmented into equal parts, then the secret message bits is inserted after each part.	High capacity	Payload not equal zero and unblinded algorithm
Complementary pair rules	It uses a defined rules by the sender to insert the data in some certain parts of the DNA.	Secure as the attacker has to know rules to extract the data	High modification rate, payload not equal zero and unblinded algorithm
Substitution	Substitute some parts of the DNA that are randomly selected	Secure, high capacity and the payload is equal to zero	Unblinded algorithm

Eventually, the substitution method is considered a secure method as the payload is zero and there are no observed changes to the DNA (Malathi et al., 2017). Additionally, it has a high capacity.

Moreover, based on the comparison between the three main methods that are accomplished by Zebari et al. (2021) the substitution method is considered the strongest because it keeps the payload constant at zero. Therefore, we will use this method in our proposed technique to embed the secret message in the DNA sequence.

To determine where the secret message will be embedded in the DNA sequence. We used the pseudo-random generator (PRG), which is an algorithm that can provide random sequences of numbers that are indistinguishable from truly random numbers. The initial value can reproduce these numbers entirely, which is the seed. Moreover, random sequences of numbers are unpredictable, so it preserves the overall security (El Hanouti et al., 2020).

### Image-based steganography Layer

As previously mentioned, there are two categories for image steganography techniques: the spatial domain and the transform domain. Table 4 shows an analysis of various techniques of steganography. Based on this analysis, we have selected the DCT method as it is highly secure, and it is challenging to detect the secret data in the image. Moreover, it is easy to implement compared with the transform domain methods (Hussain et al., 2018). Mainly, DCT divides the image into three frequency bands (low, medium, and high) that help select which band should contain the secret data. The mid-frequency DCT coefficients were chosen in order to ensure that the visual properties of the modified image are not adversely affected by the embedding effects (Evsutin, Melman & Meshcheryakov, 2020). DCT coefficient is applied using Eqs. (3), (4) and (5)) (?) where *F*(*k*, 1) is the DCT of position (*k*, 1), *M* and *N* are the row and column of image.

(3) \begin{eqnarray*}F(k,l)={a}_{k}{a}_{l}\sum _{x=0}^{M-1}\sum _{y=0}^{N-1}f(x,y)cos \frac{\pi (2x+1)k}{2M} cos \frac{\pi (2y+l)l}{2N} \end{eqnarray*}

where:

(4) \begin{eqnarray*}{a}_{k}= \left\{ \begin{array}{@{}ccc@{}} \displaystyle \sqrt{(1/M)}&\displaystyle for\,\,k=0&\displaystyle \\ \displaystyle \sqrt{(1/M)}&\displaystyle for\,\,1\leq k\leq M-1 \end{array} \right\} \end{eqnarray*}

(5) \begin{eqnarray*}{a}_{l}= \left\{ \begin{array}{@{}ccc@{}} \displaystyle \sqrt{(1/N)}&\displaystyle for\,\,k=0&\displaystyle \\ \displaystyle \sqrt{(1/N)}&\displaystyle for\,\,1\leq k\leq M-1 \end{array} \right\} \end{eqnarray*}

**Table 4:** Comparison of various techniques of image steganography.

Methods	Domain	Capacity	Imperceptibility	Robustness	Implementation
LSB	Spatial	High	High	Low	Very simple
PVD	Spatial	Medium	High	Low	Very simple
DCT	Transform	Medium	High	High	Easier than other transform domain methods
DWT	Transform	Medium	High	High	Complex
DFT	Transform	Medium	High	High	Complex

### The proposed technique: embedding mechanism

**Figure 2:**
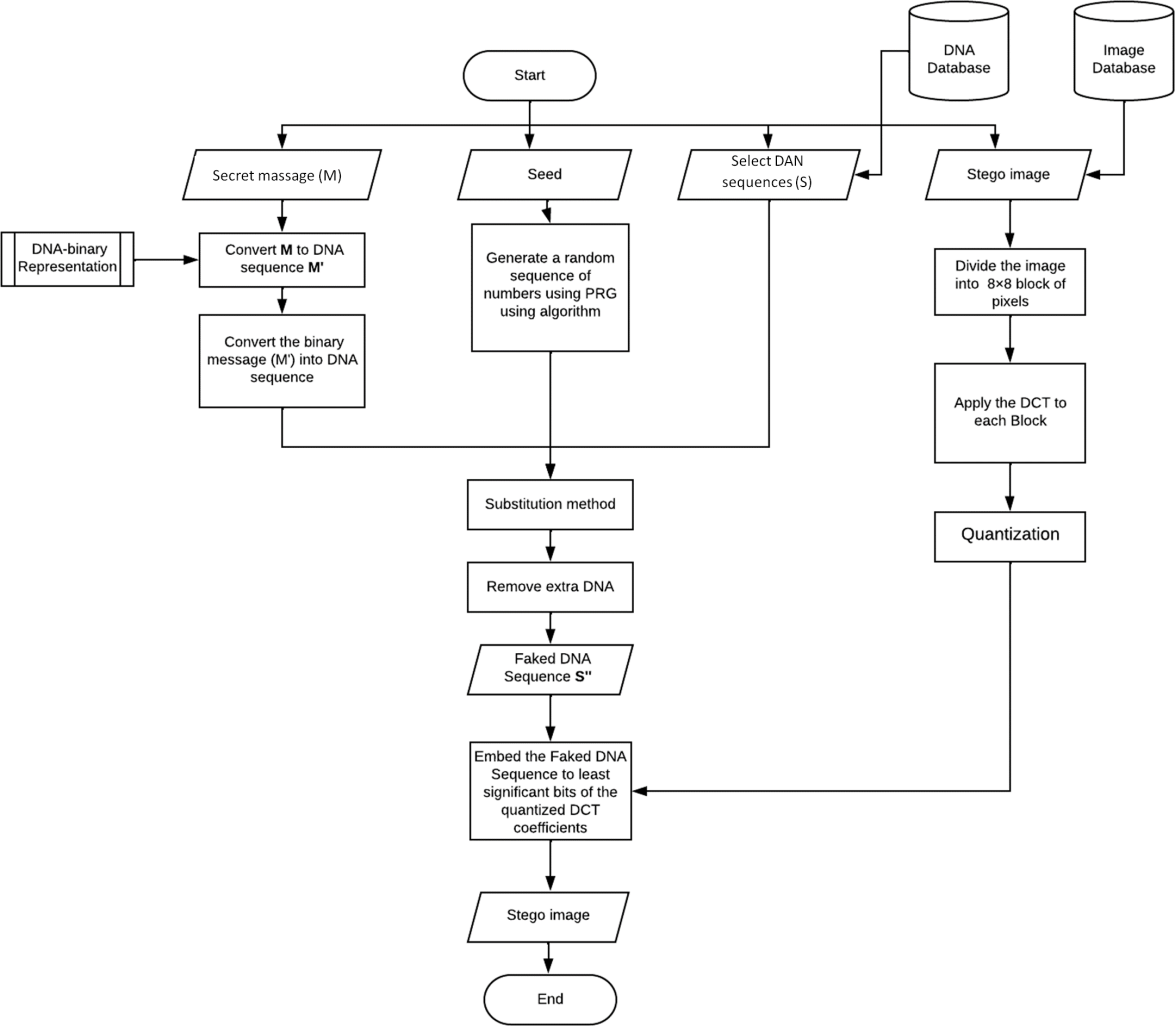
Flow chart of the embedding mechanism.

In this section, we will illustrate the embedding technique. Figure 2 shows its workflow. As mentioned before, the proposed work consists of two layers, so we will explain each one separately in the following sections.

### Layer 1: DNA steganography

1. Convert the message (M) into binary (M’) using algorithm 1.
2. Convert the binary message (M’) into a DNA sequence (*M*_*DNA*_) using algorithm 2.
3. Generate a random sequence of numbers using PRG using algorithm 3, the numbers in the sequence should be between 0 and 2.
4. Substitution method: In each segment, replace the nucleotide in the DNA sequence with a secret message nucleotide based on the location value gotten by the PRG using algorithm 4.
5. Remove extra DNA; we will keep only the used sequence part

### Layer2: image steganography

1. Dividing the original image into 8 × 8-pixel blocks.
2. In each block, DCT is applied, and the output is the DCT coefficients.
3. coefficients located in the mid-frequency range are selected in each block. The location of the two values has to be symmetric ex: D[4,5] and D[5,4].
4. For each bit in the *DNA*_*Fake*_: to transmit a 0, We will increase the first coefficient ex: D[4,5] and reduce the second ex: D[5,4]. Moreover, to transmit a 1, reduce the first coefficient ex: D[4,5] and increase the second D[5,4]. Algorithm 5 shows the pseudo code for hiding the fake DNA into the orignal image.

### The proposed technique: extracting mechanism

**Figure 3:**
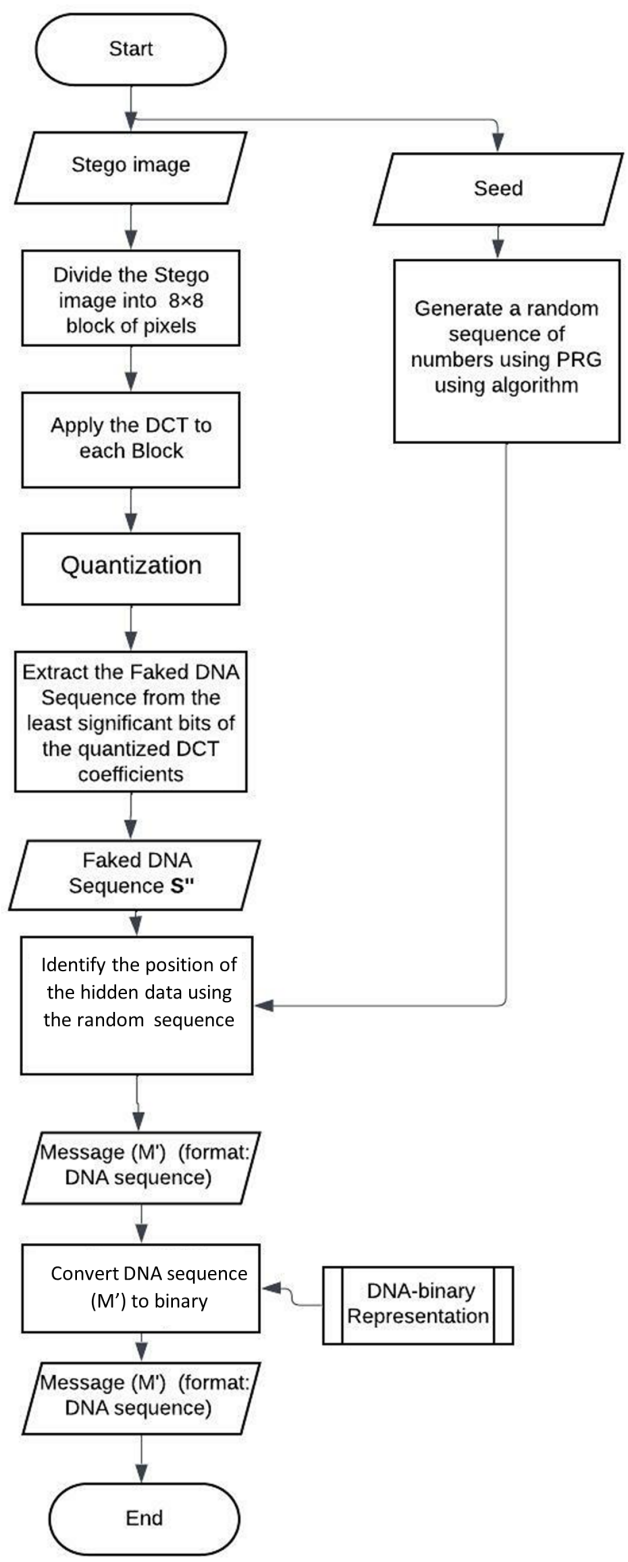
Flow chart of the extraction mechanism.

The extraction workflow is shown in Fig. 3. The extraction of the secret data from the stego image is shown in the below points.

• Dividing the Stego original image into 8 × 8-pixel blocks.
• In each block, DCT is applied, and the output is the DCT coefficients.
• Each block is compressed through a quantization table.
• Calculate the LSB of each DC coefficient.
• Generate a random sequence of numbers using PRG.
• Segment the extracted DNA and use the random sequence to extract the nucleotide.
• Convert the nucleotide into binary using the conversion rules.

## Experimental and Results

Several types of experiments were used to evaluate and compare the proposed technique with the previously reviewed techniques. These experiments are in terms of capacity, imperceptibility, and security.

### Evaluation criteria

In this section, the experiment evaluation criteria are explained. These criteria indicate the stego-quality of the images and enable comparison with other methods.

#### Capacity.

The amount of data that can embed inside the cover image without causing any noticeable visual or statistical distortion is referred to as the payload or capacity (Kaur, Bansal & Bansal, 2014). The capacity of the steganography should be maintained without impacting its imperceptibility or security. The capacity is represented by the number of characters, bits, bytes, or kilobytes.

#### Imperceptibility metrics.

The main important for steganography is ensuring the invisibility of the secret data. Several metrics, including the human visual system (HVS), mean squared error (MSE), and peak signal to noise ratio (PSNR), can be used to assess imperceptibility (Evsutin, Melman & Meshcheryakov, 2020). We calculate these metrics to evaluate the quality of the stego-image and compare it with the original image and existing techniques. Below we explain the imperceptibility metrics: The main important criterion for steganography is ensuring the invisibility of the secret data. Several metrics, including the human visual system (HVS), mean squared error (MSE), and peak signal to noise ratio (PSNR), can be used to assess imperceptibility (Evsutin, Melman & Meshcheryakov, 2020). We calculate these metrics to evaluate the quality of the stego-image and compare it with the original image and existing techniques. Below, we explain the imperceptibility metrics:

1. The human visual system (HVS)
  HVS means that the embedded data should not be obvious to the humanvision (Atta & Ghanbari, 2018). This means that the original image must be indistinguishable from the stego image. However, secret data was embedded using the proposed technique. Visually, you can see that there is no difference between the two images. As a result, the proposed technique conceals the hidden data in the original image successfully and without distortion.
2. Mean squared error (MSE)
  By measuring the distortion in the stego-image, this metric demonstrates the variation between the original image and the stego-image. By comparing each byte in the stego-image with the corresponding byte in the original image. The comparison’s result is the mean of the squared intensity differences between the two images (Shah & Bichkar, 2018). This value should be as low as possible to achieve a high-quality stego-image that looks identical to the original image. The MSE is calculated using (Eq. (6)) where the image is divided into N columns and M rows. A pixel’s value at the (i,j) location of the original image is represented by *xij* in each column, while a pixel’s value at the (i,j) position of the stego-image is represented by *yij* (Kaur & Rani, 2016).
  (6) \begin{eqnarray*}MSE= \frac{1}{(MN)} \sum _{i=1}^{M}\sum _{j=1}^{M}({x}_{ij}-{y}_{ij})^{2}\end{eqnarray*}
3. Peak signal to noise ratio (PSNR)
  The most common metric is PSNR. By dividing the highest color intensity value in the cover image by the MSE, it determines the stego-quality. A large value of PSNR means less distortion; therefore, there is a small difference between the original and stego images (Kaur & Rani, 2016). The PSNR is calculated using (Eq. (7)).
  (7) \begin{eqnarray*}PSNR=10\cdot {\log \nolimits }_{10} \frac{(255)^{2}}{(MSE)} \end{eqnarray*}
4. Structural Similarity Index (SSIM)
  The SSIM is another metric for measuring the similarity between the cover and stego image in terms of structural information changes by modeling the image into three elements: the loss of correlation, the luminous distortion, and the contrast distortion. It is used to quantify image quality distortion after embedding the secret data (Hashim et al., 2018). The outcome of SSIM is a numerical value between zero and one. “1” means that the stego image and original are a perfect match, which means if the outcome value is close to “1” then the stego image has less distortion. There is, therefore, a very small and non-noticeable difference between the original and stego. The SSIM is calculated using (Eq. (8)). where *μ*_*C*_ and *μ*_*C*∗_ are the average of original image (C) and the stego image (C*), ${\sigma }_{C}^{2}$ and ${\sigma }_{C\ast }^{2}$ are the variance of C and C*. And, *μ*_*C*_*μ*_*C*∗_ is the co-variance of C and C*, and Divide by *d*_1_ and *d*_2_ which are two variables used to stabilize the division with a weak denominator (Pan et al., 2022).
  (8) \begin{eqnarray*}SSIM= \frac{({\mu }_{C}{\mu }_{C\ast }+{d}_{1})({\sigma }_{CC\ast }+{d}_{2})}{({\mu }_{C}^{2}+{\mu }_{C\ast }^{2}+{d}_{1})({\sigma }_{C}^{2}+{\sigma }_{C\ast }^{2}+{d}_{2})} \end{eqnarray*}
5. Image Fidelity (IF)
  This metric determines the general image quality in terms of visual equivalent. The outcome is a numerical value between zero and one. One means that the stego image and original are a perfect match and have less distortion. There is, therefore, a very small and non-noticeable difference between the original and. It is calculated using (Eq. (9)) where *C*_*t*_ and *C*_*i*_ are cover bits and stego bits, respectively, and *H* × *W* is the image size measured in bits per pixel (Elkandoz, Alexan & Hussein, 2019).
  (9) \begin{eqnarray*}IF=1- \frac{\sum _{t=1}^{H\times W}({C}_{t}-{S}_{t})^{2}}{\sum _{i=1}^{H\ast W}({C}_{i})^{2}} \end{eqnarray*}
6. Entropy
  This metric is used to measure the amount of information that the stego image contains compared to its original image. Through calculating the difference in uncertainty between both images Thus, the higher this value, the more information the image contains. Entropy is calculated using (Eq. (10)) Where in each color channel, the set of gray levels in that channel is represented by N, and their probability is given by p(x) (Elkandoz, Alexan & Hussein, 2019).
  (10) \begin{eqnarray*}Entropy=\sum _{x=0}^{N-1}p(x){\log \nolimits }_{2}p(x)\end{eqnarray*}

#### Security analysis.

The essential requirement for steganographic techniques is resistance against unauthorized attacks. This measure is known as robustness. Usually, attackers attempt to retrieve or detect the presence of secret information inside the stego-image (Kaur & Singh, 2021). To evaluate the security, we used the two most well-known steganography attacks, which are the histogram analysis and the chi-square attack. These attacks are regarded as one of the most effective experiments for assessing the security of a stego-image. The following sections illustrate them:

1. Histogram analysis
  Histogram analysis is a mathematical analysis of the image data used to identify the correlation between the cover image and the stego image. It is a graphical diagram in which the *x*-axis and *y*-axis explain the pixel difference between each pair of pixels and the number of occurrences, respectively (Pradhan et al., 2016). Moreover, it visualizes the differences in pixel value level between the cover image and its corresponding value in the stego images. These differences can be easily observed.
2. Chi-square attack
  The chi-square is another well-known method that is used to determine the robustness of the steganography technique against attacks by checking the difference of statistical probability analysis between the original image and the stego image that contains the secret data. The outcome of this method is a numerical value between 0 and 1 (Hosam, 2019). If the difference is close to one, it indicates that no data is hidden within the image; otherwise, it indicates that the image contained secret data.

### Experimental setup

Experiments were developed using the Conda environment in a Jupyter notebook and the Python language. With the help of several libraries like: *numpy* for the advanced calculations, *skimage* metrics for imperceptibility metrics, *matplotlib* for histogram analysis and CV2 (*HISTCMPCHISQR*) for chi-square.

#### Secret Message.

Different plain text is used in testing with different sizes. All the plain texts contain letters, numbers, and special characters to cover all the possibilities of the plain text.

#### Images.

To evaluate the proposed technique, different images of different types and sizes were used. These image types are BMP, PNG, and JPG with two different dimensions: 1,024 × 1,024 and 512 × 512 pixels. These images were chosen from the SIPI image database, https://sipi.usc.edu/database/.

USC-SIPI image database is divided into four volumes based on the pictures’ characters. For the testing, we have used six color images with different extensions and dimensions. The experiment runs using these images while using different payloads (20, 30, 40, 50, 60, 70, 80, and 90).

#### DNA Sequences.

Different DNA sequences with different sizes are used to hide the plaintext. The below table, Table 5 gives the DNA sequences and their scientific names, as well as the number of nucleotides in the DNA. These DNA samples were collected from the National Center for Biotechnology Information: https://www.ncbi.nlm.nih.gov/.

### Experimental results

#### Capacity results.

As we are using the DCT to hide the data in the images, the maximum capacity is limited by the number of quantized DCT blocks in the image. Table 6 shows the image capacity in terms of the maximum characters and bits that can be embedded in each image.

The results show that the proposed algorithm can hide a large amount of data, up to 90% of the image size, whatever the image type. Also, the maximum capacity is 1,024 characters (8,192 bits) using 1,024 image size. This means the maximum capacity is equal to the image size for the 1,024 image size.

#### Imperceptibility metrics results.

Table 7 shows the MSE, PSNR, SSIM, IF, and Entropy for the images used in the evaluation and comparison between the original and stego image after the embedding procedure. In addition, the results show that there are no visible artifacts between the two images when viewed visually using the HVS. Hence, the proposed technique successfully hides the secret data in the original image without any distortion.

**Table 5:** DNA sequences used in testing.

Locus	Nucleotide	Common name
DP001042.1	174523	Rabbit
DP001046.1	173940	Black-handed spider monkey
AC254805.6	147388	Dog
AC254809.1	127817	Human
AC254802.6	144410	Dog

**Table 6:** The maximum capacity per image.

Image type	Image size	Capacity (Char)	Capacity (Bits)
BMP	512	188	1504
	1024	1024	8192
JPG	512	256	2048
	1024	1024	8192
PNG	512	512	4096
	1024	1024	8192

**Table 7:** Imperceptibility metrics values at different payloads.

Type	Size	Payload	MSE	PSNR	SSIM	IF	Entropy
PNG	512	10	0.011	67.708	0.999981	0.9995	0.002
		50	0.032	63.047	0.999953	0.99832	0.009
		90	0.078	59.234	0.999889	0.9961	0.017
PNG	1024	10	0.004	72.217	0.999936	0.99997	0
		50	0.008	69.156	0.9999	0.99968	0.001
		90	0.012	67.502	0.999887	0.99935	0.001
JPG	512	10	11.413	37.557	0.98144	0.7431	0.081
		50	11.414	37.557	0.981437	0.74309	0.081
		90	11.413	37.557	0.981438	0.7431	0.081
JPG	1024	10	46.695	31.438	0.888436	0.49025	0.018
		50	46.702	31.437	0.888428	0.49023	0.018
		90	46.704	31.437	0.888422	0.49024	0.018
BMP	512	10	0.009	68.422	0.999958	0.99936	0
		50	0.042	61.885	0.999847	0.99634	0.003
		90	0.064	60.052	0.999766	0.99419	0.007
BMP	1024	10	0.002	76.252	0.999976	0.99991	0
		50	0.006	70.107	0.999939	0.99951	0.004
		90	0.011	67.845	0.999912	0.99911	0.007

The results show that the MSE, PSNR, SSIM, IF, and Entropy values for PMP and PNG are excellent, as the minimum PSNR value is 60.052 and the MSE is 0.064, which means distortion is exceptionally minimal. On the other hand, we found that JPG images are not sufficient for hiding the data using the proposed technique, as the PSNR value is between 37 and 31, and the MSE is between 11.413 and 46.704. These ratios affect the quality of the image.

Moreover, the values for SSIM and IF for the PMP and PNG are excellent as the minimum SSIM value is 0.999766 and IF is 0.994189, which are very close to one, which means that the stego image and original are a perfect match and have less distortion. On the other hand, the SSIM and IF values for JPG are lower than for other image types. The value of the SSIM is between 0.888422 and 0.98144 and the IF value is between 0.490219 and 0.743097. These ratios affect the quality of the image.

Furthermore, the entropy metric determines the amount of information the image contains. A higher value may lead to the exposure of hidden data. The average entropy values of PMP and PNG are very good, as BMP’s average value is 0.005 and PNG’s is.004, which are near zero. Contrarily, the JPG images had a higher value, which is 0.81, and this ratio will expose the existence of hidden data.

#### Security analysis result

In the histogram, the values of the cover and stego-image are calculated, and the histogram for each image is drawn. Then, it’s compared to identify the distortion between the images. Table 8 shows the histogram analysis result. Clearly, it can be seen that the PNG and BMP histograms are nearly identical, at variance with the JPG histograms.

**Table 8:** Comparison between the histogram of original image and the stego image.

**Type**	**Size**	**Payload**	**Histogram of original image**	**Histogram of stego image**
PNG	512	10	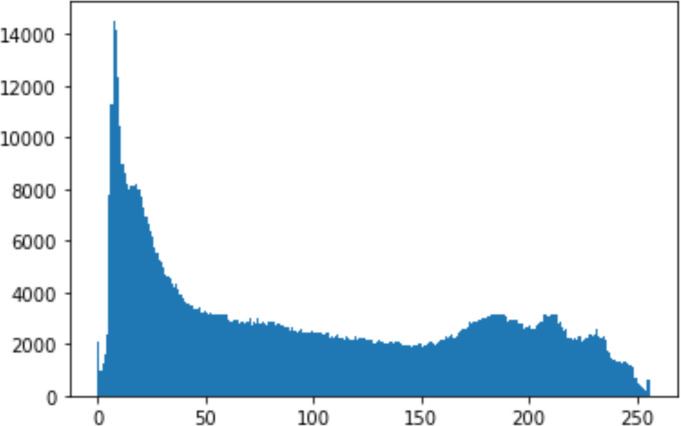	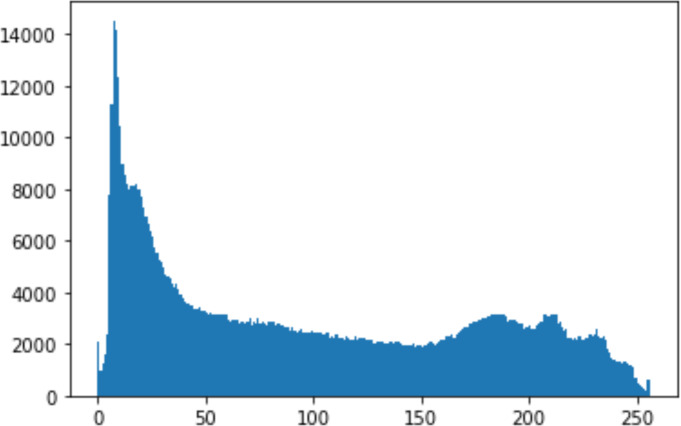
		50	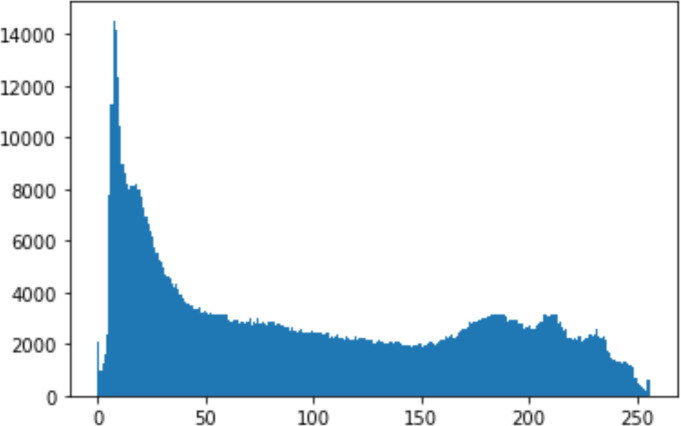	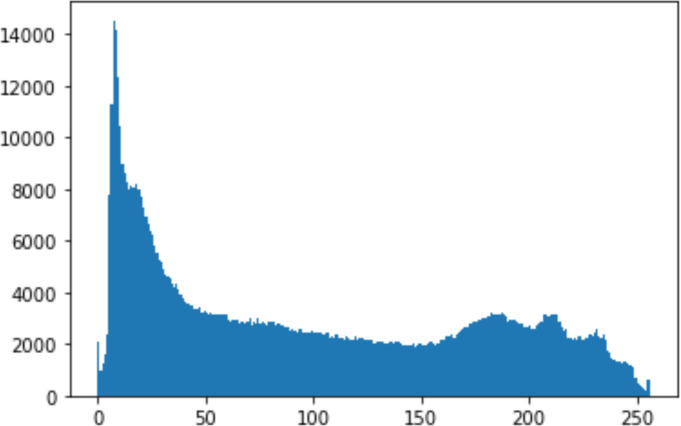
		90	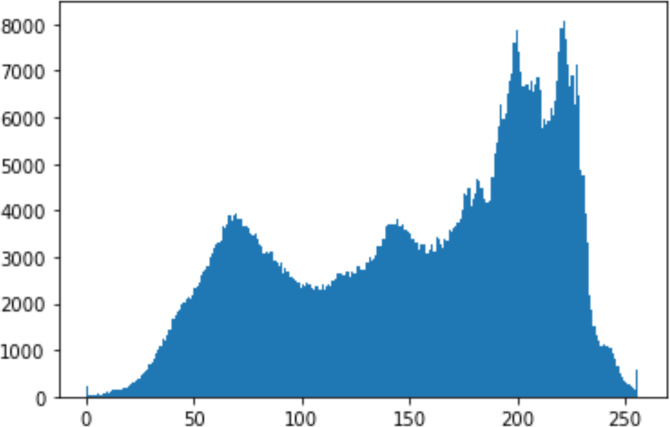	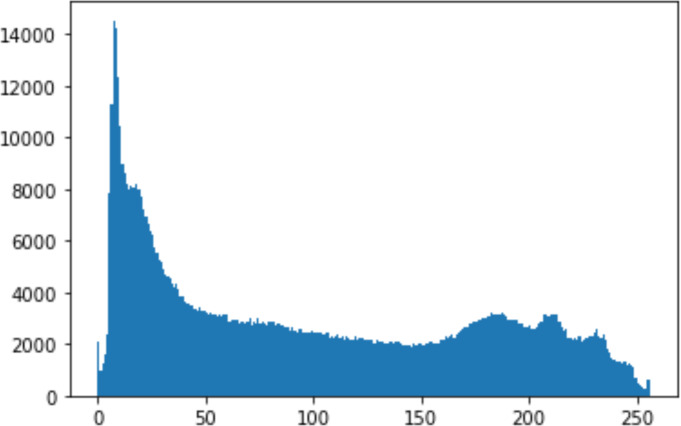
PNG	1024	10	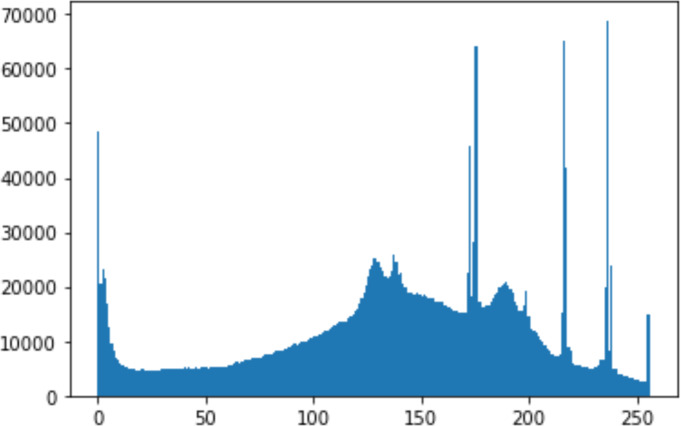	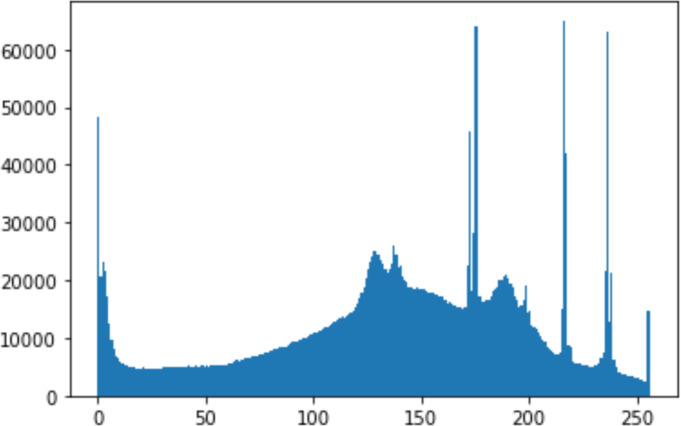
PNG	512	50	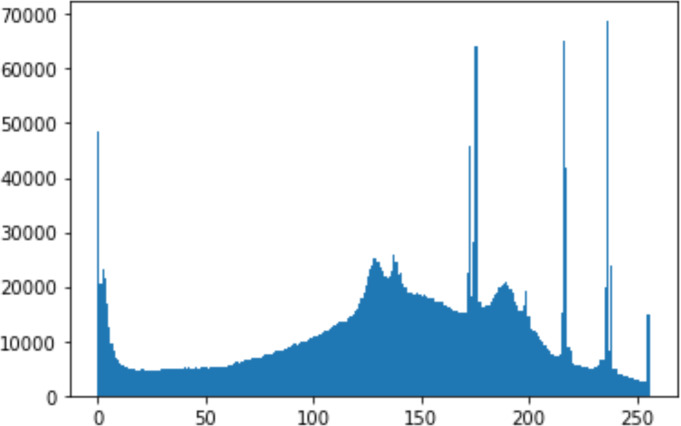	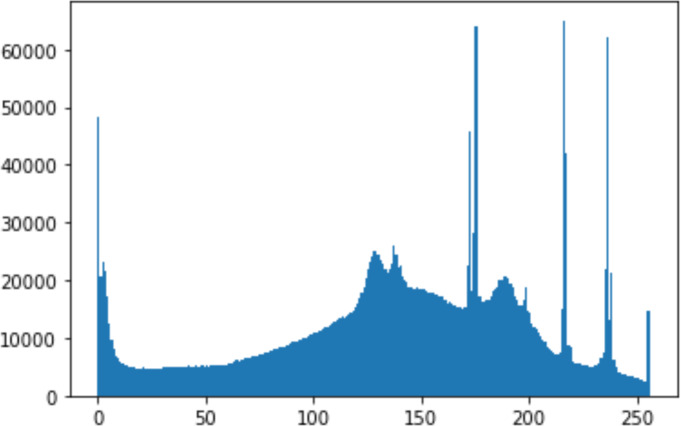
		90	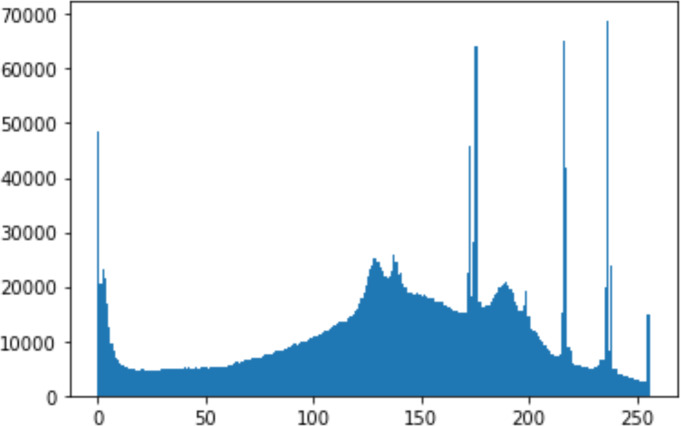	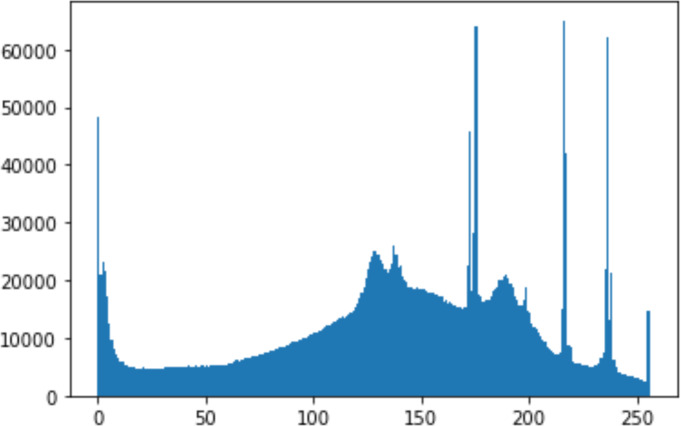
JPG	512	10	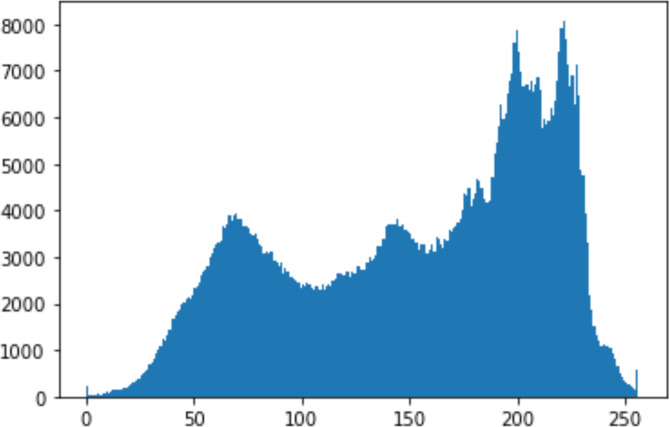	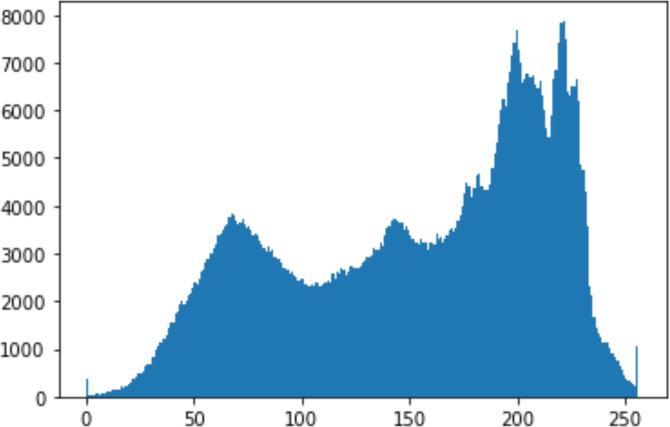
		50	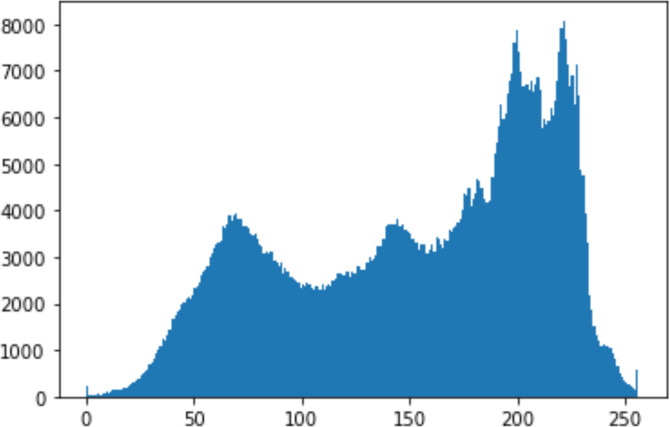	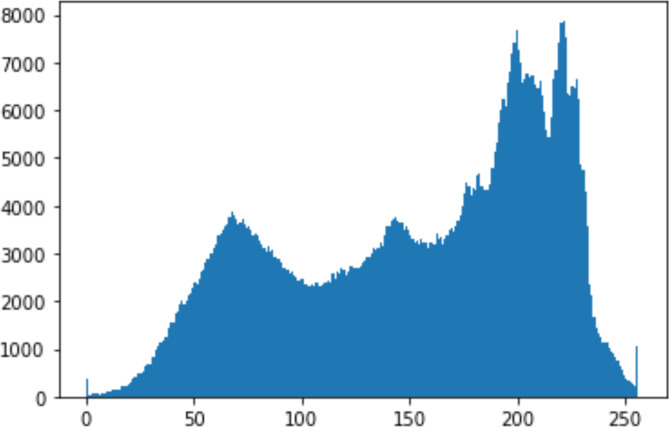
		90	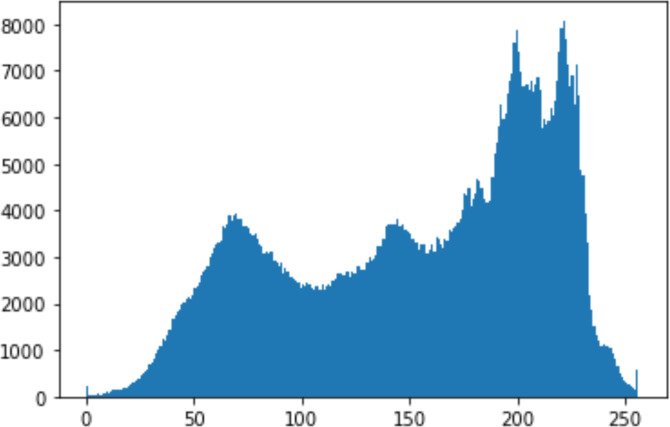	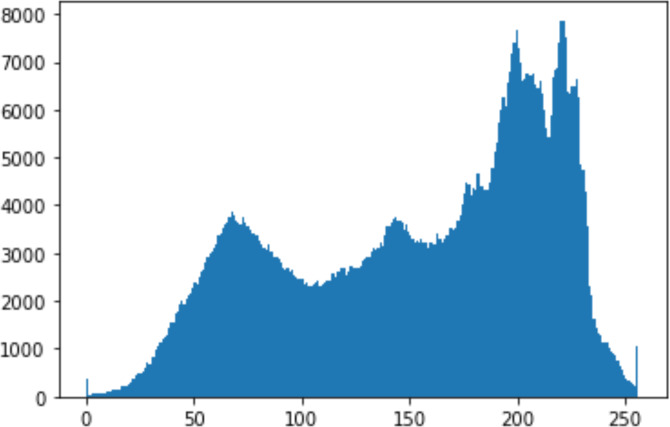
JPGn	1024	10	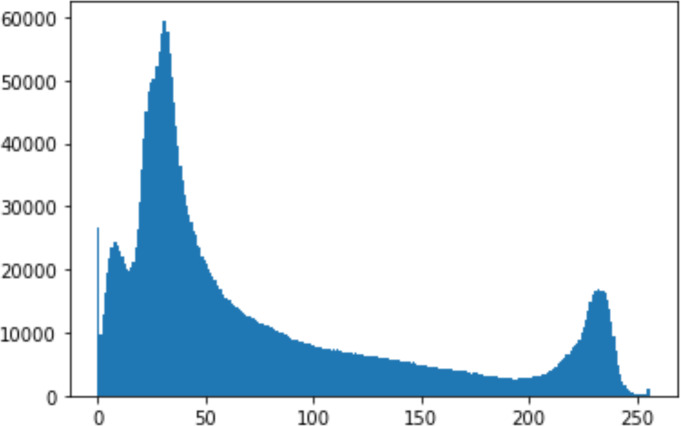	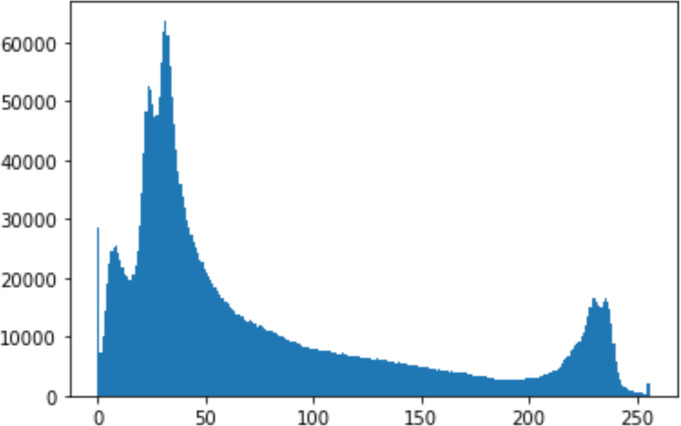
JPG	1024	50	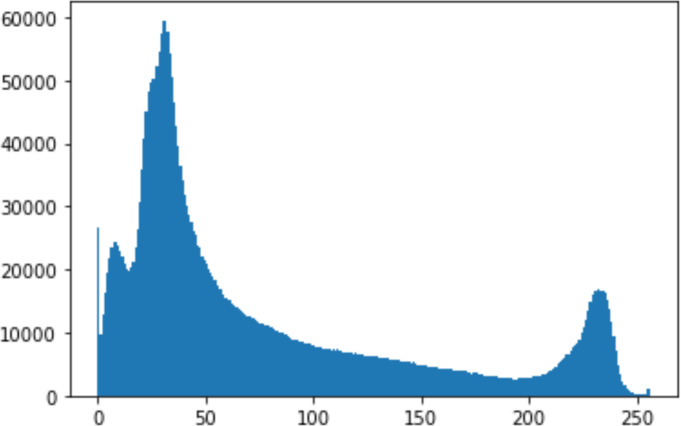	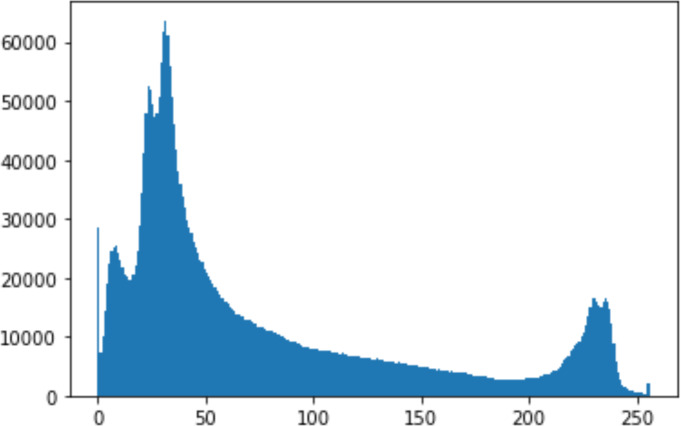
		90	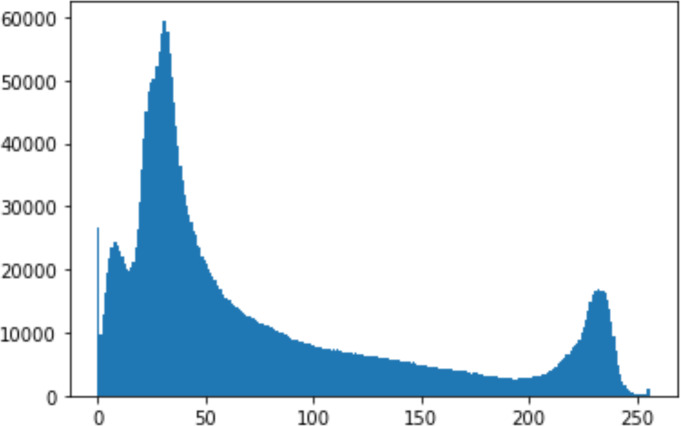	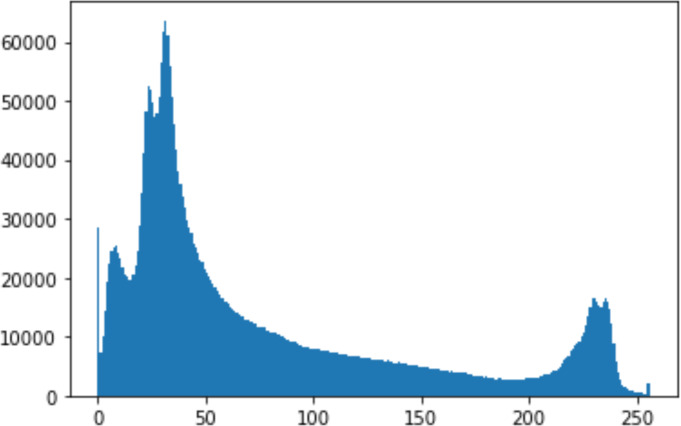
BMP	512	10	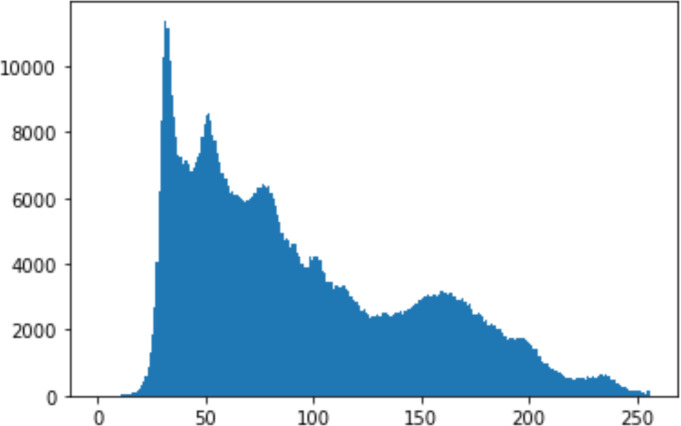	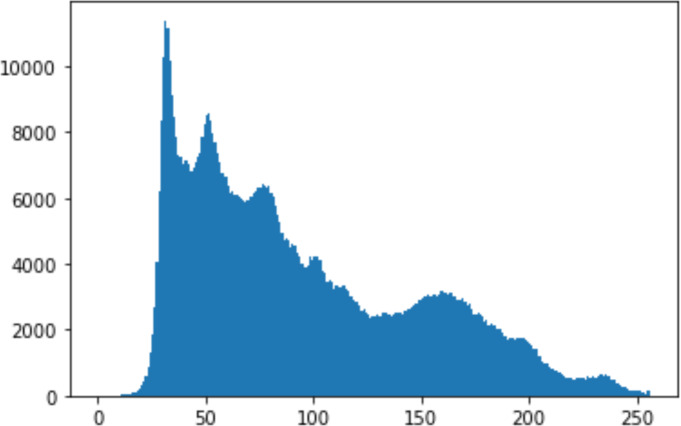
		50	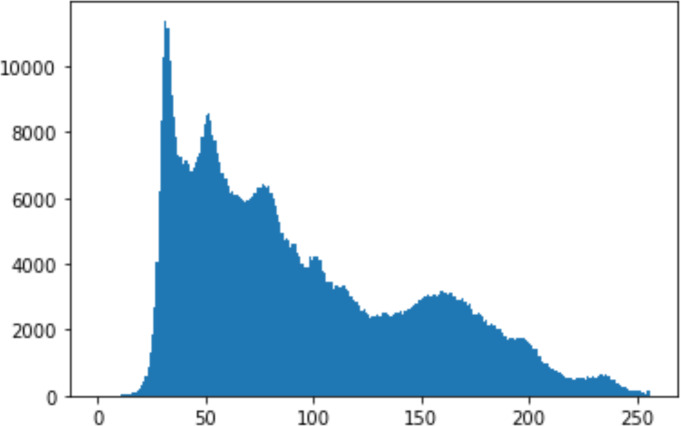	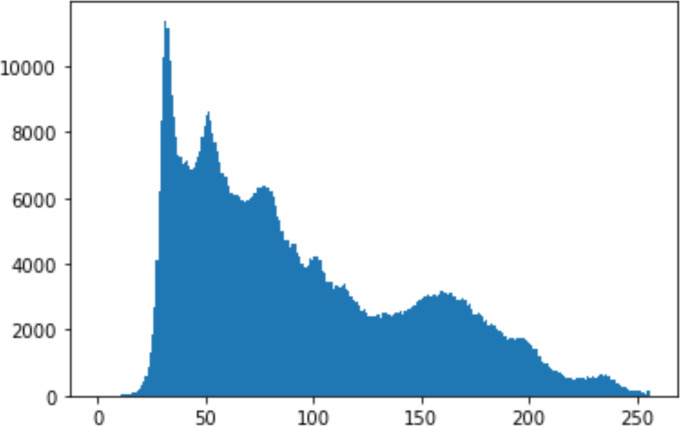
		90	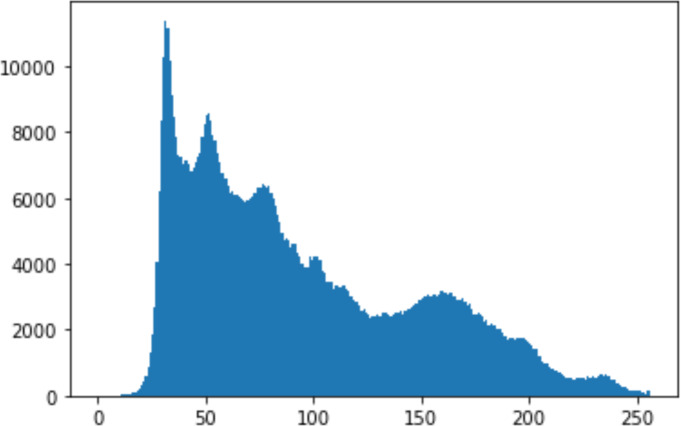	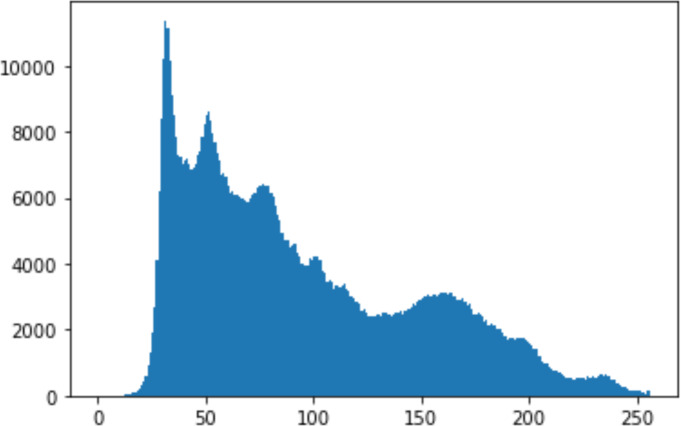
BMP	1024	10	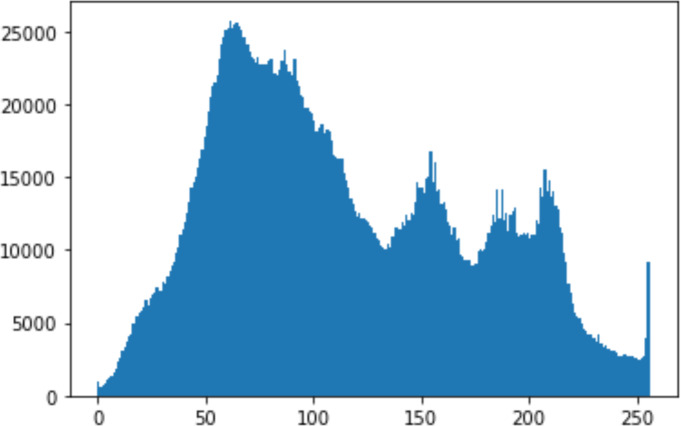	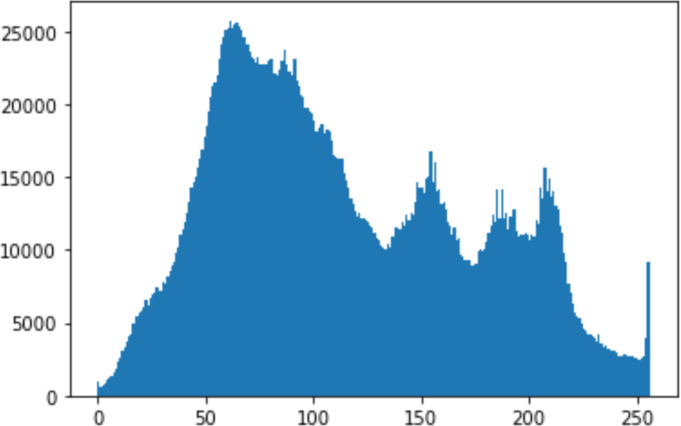
BMP	1024	50	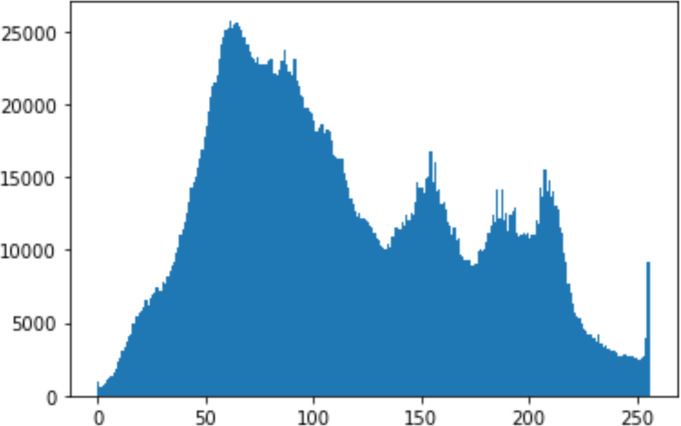	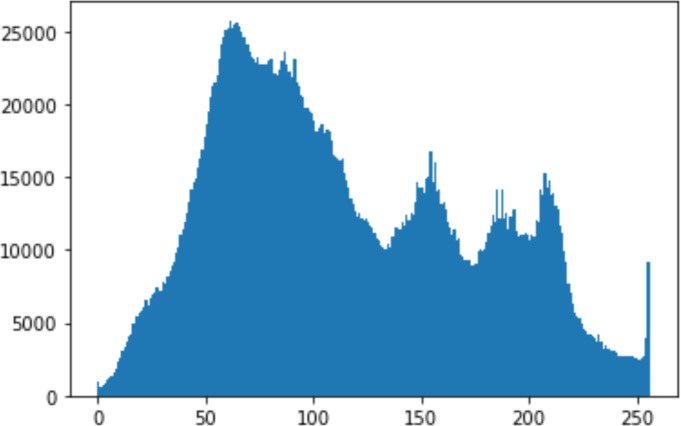
		90	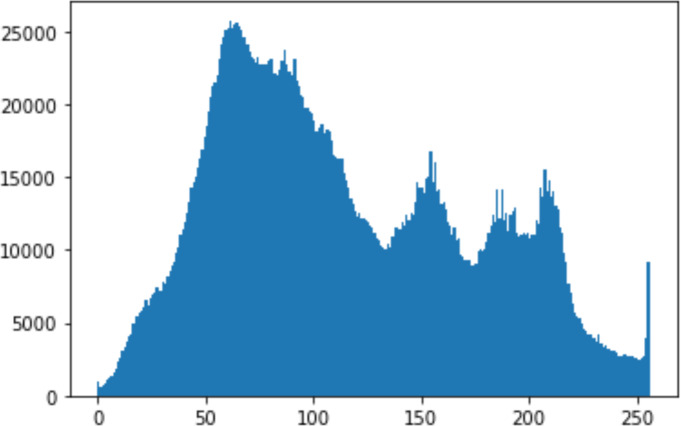	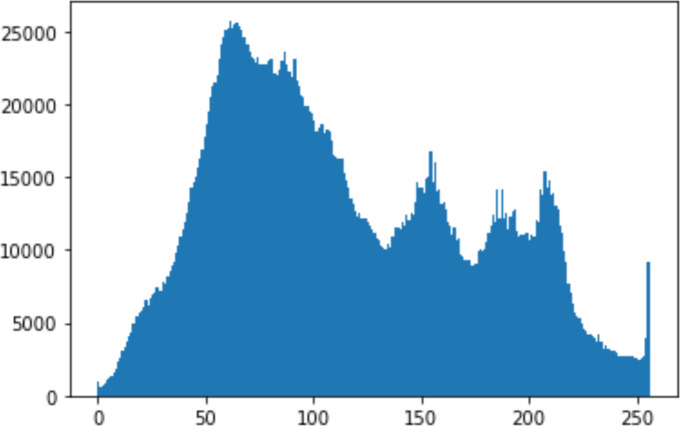

From the histogram, we obtained the mean, standard deviation, and variance to identify the differences between the two histograms. See Table 9. From these values, we can see that the BMP and PNG images have the lowest values. Meanwhile, the JPG has higher values.

**Table 9:** Comparison between the histogram of the original image and stego image in term of mean, varanse and standred devation.

**Type**	**Size**	**Payload**	**Mean**	**Variance**	**Standard deviation**
PNG	512	10	0.01	0.55	0.00
		50	0.01	1.69	0.01
		90	0.03	4.07	0.03
	1024	10	0.00	0.05	0.00
		50	0.00	0.04	0.00
		90	0.00	0.09	0.00
JPG	512	10	0.41	11.78	0.08
		50	0.41	11.70	0.08
		90	0.41	11.67	0.08
	1024	10	0.30	32.50	0.26
		50	0.30	33.10	0.26
		90	0.30	33.23	0.26
BMP	512	10	0.00	0.00	0.00
		50	0.00	0.03	0.00
		90	0.00	0.23	0.00
	1024	10	0.01	0.79	0.01
		50	0.02	1.33	0.01
		90	0.02	1.80	0.01

In addition, probability analysis of the Chi-Square security metric for the cover and stego-image is calculated, as shown in Table 10. For the BMP and PNG images, the values are around zero, which does not cause any suspicion regarding the existence of secret information. On the other hand, the PNG values are higher.

**Table 10:** Chi-Square Security Metric Values.

**Type**	**Size**	**Payload**	**Chi-Square**
PNG	512	10	0.001
		50	0.014
		90	0.054
	1024	10	0.001
		50	0.001
		90	0.001
JPG	512	10	0.709
		50	0.711
		90	0.71
	1024	10	0.149
		50	0.157
		90	0.159
BMP	512	10	0.00
		50	0.002
		90	0.01
	1024	10	0
		50	0.003
		90	0.007

As a conclusion, the PNG and BMP images successfully passed the histogram and Chi-Square attacks.

## Comparison and Discussion

This section is to discuss and compare the proposed technique with the previously reviewed techniques in terms of the hiding mechanism, amount of information to be hidden, security and robustness, and finally the imperceptibility metrics.

### Capacity

The amount of data to be hidden depends on the image size because we are using the DCT approach to hide the data. Thus, the maximum capacity depends on the number of 8 × 8 quantised DCT blocks in the images. Also, the previously reviewed techniques used methods that hid the data in the spatial domain, which has a high capacity compared with the frequency domain that we are using in the proposed technique. The below Table 11 shows a comparison between the techniques using 1,024×1,024 image. And as seen, the proposed technique’s capacity is over 20% less than the other ones shown on Table 11.

**Table 11:** Capacity comparison between the proposed technique and existing techniques.

Techniques	Capacity
Das et al. (2015)	10,922 bits
Tuncer & Avci (2016)	10,922 bits
Proposed technique	8,192 bits

### Imperceptibility metrics

The proposed method is compared with Vinodhini & Malathi (2018), Das et al. (2015), Tuncer & Avci (2016) and Durafe & Patidar (2022) in terms of average MSE and PSNR in Table Table 12. The MSE results of the proposed technique are 0.022889 for the BMP image, 0.022833 for the PNG image, and 29.05744 for the JPG image. The MSE values for the three studies compared are 0.172449, 0.203033, null, and 0.001 respectively.

**Table 12:** A comparison between the proposed technique and existing techniques in terms of MSE and PSNR.

**Metrics**	**MSE**	**PSNR**
**Vinodhini & Malathi (2018)**	0.172449	55.724
**Das et al. (2015)**	0.203033	55.85522
**Tuncer & Avci (2016)**	Null	52.195
**Durafe & Patidar (2022)**	0.001	86.15
**Proposed technique**	BMP = 0.022889 PNG = 0.022833 JPG = 29.05744	BMP = 66.85733 PNG = 66.28528 JPG = 34.49706

The proposed method has the following PSNR values: BMP = 66.85733, PNG = 66.28528, and JPG = 34.49706, whereas the three compared studies have 55.724, 55.85522, 52.195, and 86.15 respectively.

Comparing these values to the ones given by the above-mentioned studies, the results show clearly that the values of the proposed technique using BMP and PNG are higher than the majority , which means the proposed technique has good concealment.

Moreover, in Table 13, the entropy values were compared with Das et al. (2015). The results show that the values of the proposed technique using BMP and PNG are higher than those of the other technique.

**Table 13:** A comparison between the proposed technique and existing techniques in term entropy.

Metrics	Das et al. (2015)	Proposed technique		
		BMP	PNG	JPG
Entropy	0.203033	0.022889	0.022833	29.05744

### Robustness to attacks and Security

The results of the security analysis of our techniques were compared with previously reviewed techniques. Table 14 shows the mean and standard deviation. We can see that our technique has good value compared with the other techniques. In addition, the DCT possesses resistance to signal processing operations like noise attack and compression since it belongs to the frequency domain methods Lin, Shie & Guo (2010).

**Table 14:** A security comparison using histogram analysis between the proposed technique and existing technique.

Technique	Security analysis	Mean	Standard deviation
Das et al. (2015)	Cover Histogram	145.7929	76.3373
	Stego Histogram	145.795	76.332
	Differences	0.0021	0.0053
Proposed technique	Cover Histogram	98.2571	69.365
	Stego Histogram	98.2557	69.3648
	Differences	0.0013	0.00024

The substitution technique is used, as was already indicated, to conceal the secret data inside the cover image. And in the field of DNA steganography, this method is the most reliable one compared with others. The proposed method is hence resistant to attacks for the following reasons:

• There are many databases providing access to more than 163 million DNA sequences Shiu et al. (2010). As a result, there is a low likelihood of detecting the original DNA; thus, it becomes challenging to determine whether it contains any concealed secret information. In addition, the probability of identifying the original sequence is $ \frac{1}{1.63\ast 1{0}^{8}} $
• Since PRG is used with the substitution method, it becomes harder for the attacker to identify how the secret message was concealed in the DNA sequence. We propose, as future work, to incorporate cryptographically secure PRG (CSPRG) for significantly improving our methods.

### Execution time

The average execution time of the proposed system is 0.6 s, computed using a Jupyter notebook on an Intel Core i5 processor having 8 GB, which is less than the required time used in the DWT-SVD (1.83 s) and IWT-SVD scheme (1.48 s) presented in Durafe & Patidar (2020). However, the execution time varies and depends on the complexity of the algorithm and the hardware used for testing.

## Conclusion

This work aims to create a robust double-layer steganography technique by hiding sensitive information in a DNA sequence and then hiding the DNA sequence inside an image. 

We chose to use DNA steganography because of its complexity and randomness, which offer high protection, security, and robustness.

Also, we chose to hide the DNA sequence to guarantee the invisibility of sensitive information and avert attackers’ suspicion.

The implementation was done using Python and the Jupyter notebook. We used the substation method with the help of the PRG to hide the data inside the DNA technique. We used the DCT to hide the DNA sequence inside the image.

During the evaluation of the proposed technique, we used different types of images with different sizes, along with different lengths of messages, and DNA sequences were used during the experiment.

The proposed technique proved its ability to hide sensitive data in different image formats, like PNG, JPG, and PMP, with different sizes. Also, it has the capability to hide varied message lengths, up to 1,024 characters. As shown before, there was no visual distortion between the cover and stego images.

In terms of security, it is proven that it persists against attacks, as well as its use of DNA as a first cover layer, which gives a double layer of security.

The result showed that the ratios of imperceptibility metrics for PNG and BMP were outstanding, and they outperformed the existing systems. Contrarily, the JPG images failed to meet the perfect ratios. Moreover, our technique has less capacity than others because we use DCT to hide the data in the image. However, the DCT grants a very secure mechanism for hiding the data in the images. Moreover, in the security analysis, our technique using PNG and BMP has sufficiently good values and is outperforming the existing systems. In the future, more research can be done to evaluate the proposed technique’s performance and robustness with respect to image rotation, scaling, or cropping. In addition, as future work, we propose incorporating cryptographically secure PRG (CSPRG) to significantly improve our methods.

## Supplemental Information

10.7717/peerj-cs.1379/supp-1 Supplemental Information 1 Code used to implement and test the proposed slution https://github.com/OmniaAlharbi1/Double-Layer-Steganography-Technique-using-DNA-Sequences-and-Images.git

## Additional Information and Declarations

### Data Availability

The following information was supplied regarding data availability: The code is available in the Supplemental File, GitHub and Zenodo:
https://github.com/OmniaAlharbi1/Double-Layer-Steganography-Technique-using-DNA-Sequences-and-Images.git
Alharbi, Omnia. (2022). Steganography Technique Using DNA Sequences and Images (2.0.4). Zenodo. https://doi.org/10.5281/zenodo.7046304. The data is available at the NCBI: NC_048221.1, NC_048236.1, CM039651.1, CM039643.1, CM026147.1, OU342652.1
The Image Database is available at the USC Signal and Image Processing Institute: https://sipi.usc.edu/database/database.php?volume=misc.
